# Fitness costs associated with unnecessary virulence factors and life history traits: evolutionary insights from the potato late blight pathogen *Phytophthora infestans*

**DOI:** 10.1186/1471-2148-10-283

**Published:** 2010-09-16

**Authors:** Josselin Montarry, Frédéric M Hamelin, Isabelle Glais, Roselyneère Corbi, Didier Andrivon

**Affiliations:** 1 INRA, Agrocampus-Ouest, UMR1099 BiO3P (Biology of Organisms and Populations applied to Plant Protection), F-35653 Le Rheu, France; 2 INRA, UR407 Pathologie Végétale, F-84143 Montfavet, France

## Abstract

**Background:**

In gene-for-gene models of plant-pathogen interactions, the existence of fitness costs associated with unnecessary virulence factors still represents an issue, both in evolutionary biology and agricultural sciences. Measuring such costs experimentally has proven difficult, especially in pathogens not readily amenable to genetic transformation, since the creation of isogenic lines differing only by the presence or absence of avirulence genes cannot be achieved in many organisms. Here, we circumvented this difficulty by comparing fitness traits in groups of *Phytophthora infestans *isolates sharing the same multilocus fingerprint, but differing by their virulence/avirulence spectrum.

**Results:**

Fitness was assessed from calculations derived from the basic reproduction number, combining several life history traits (latent period, spore density and lesion growth rate) evaluated on leaflets of the potato cultivar Bintje, which is free of resistance genes. A statistically significant fitness cost was found in isolates virulent to the R10 resistance gene. That cost was due to a lower spore production in virulent isolates; however, the latent period was shorter in virulent isolates. Similar trends, although not statistically significant, were observed for the other genes tested.

**Conclusion:**

The data likely reflect the adaptive response of the pathogen to the cost associated with virulence. They suggest strong trade-offs between life history traits related to pathogenicity and adaptive biology of pathogens.

## Background

Costs of adaptation play a major role in host-parasite coevolution, and thus take a central place in evolutionary theory. Many plant parasites interact with their hosts according to the widely accepted gene-for-gene model, which predicts that successful disease resistance is triggered only if a resistance (R) gene product in the host plant recognizes, directly or indirectly, a specific avirulence (Avr) gene product from the pathogen [1,2]. In such systems, where resistance and pathogenicity (here termed virulence, according to Vanderplank [3]) are inherited as single genes [4], there are many recorded examples of directional selection leading to invasion of pathogen populations by virulent isolates, and hence resistance breakdown [e.g. [3,5]]. However, such invasion has been slow in other cases [6,7], suggesting that mutation from avirulence to virulence can have different fitness consequences depending on genes and populations.

A mutation from avirulence to virulence is always associated with an increase in fitness (*i.e*. the combined ability of an organism to survive and reproduce [8]) provided the R gene is present in at least part of the host population, because the virulent pathogens will be the only ones to exploit that fraction of the host population. However, mutation in an avirulence gene may also imply fitness costs, for instance if virulent pathogens perform less well (*i.e*. have a lower aggressiveness, or quantitative pathogenicity - see Pariaud et al. [9]) on hosts without the R-gene. Vanderplank [10] advocated this mechanism to explain that polymorphism for virulence can be maintained in pathogen populations exposed to hosts with R genes. Recent work [11] has shown that a cost of virulence increases the frequency of the corresponding resistance, creating frequency-dependent selection acting directly on host resistance or pathogen virulence, and would thus increase the value of the resistance gene in plant breeding.

An argument to explain that parasites would keep a molecule that allows them to be recognized by the host, and thus work as an avirulence effector, is that Avr proteins perform some essential function, and therefore must be retained [12]. Avirulence genes from plant pathogenic viruses, bacteria and fungi do not share any strong structural homogeneity, but encode very diverse proteins with a range of demonstrated or postulated physiological functions [13-15]. Many of these genes are involved in life history traits which have a measurable effect on fitness, so that their mutations can impose a cost to virulence [16].

Fitness costs associated with unnecessary virulence factors were indeed detected in several plant pathogens. For instance, virulent strains of *Xanthomonas oryzae *pv. *oryzae*, obtained through mutagenesis, caused shorter lesions than the wild-type (avirulent) strain on a rice line containing no resistance genes [17]. Similarly, a TuMV (Turnip Mosaic Virus) isolate avirulent on *TuRB01 *out-competed virulent isolates in co-inoculation experiments in a susceptible host background [18], suggesting a fitness cost to TuMV isolates overcoming resistance genes of *Brassica *crops. Detection of virulence fitness cost is possible in both agricultural and wild plant pathosystems, and in both controlled inoculation experiments and field surveys. For instance, Huang et al. [19] demonstrated that ascospores of the Brassica blackleg pathogen *Leptosphaeria maculans *avirulent to *RLm4 *produced more and larger lesions than virulent ascospores (*avrLm4*) on leaves of two *Brassica napus *cultivars lacking the corresponding resistance gene *RLm4*, and related that observation to the increasing frequency of *AvrLm4 *in two consecutive seasons of field experiments. In a similar way, Thrall and Burdon [20] described a trade-off between spore production and virulence in wild populations of the flax rust fungus *Melampsora lini*, so that selection favors virulent strains of *M. lini *in resistant *Linum marginale *populations and avirulent strains in susceptible populations. However, in many other cases, the detection of measurable fitness costs to virulence has been difficult or impossible [16], which suggests either that such costs can be low in some pathogens, or that these pathogens evolved compensatory mechanisms able to restore the fitness of virulent genotypes.

Two main approaches have been taken to evaluate the fitness cost imposed on pathogen isolates by mutation to virulence: to determine from population surveys whether virulent isolates decrease in frequency in the absence of the corresponding R-gene [21,12], or to directly determine if inactivation of an avirulence gene implies a fitness penalty [22]. Here, we used a third approach, similar to that of Bahri et al. [23] and consisting in comparing aggressiveness components in a near-isogenic collection of *Phytophthora infestans *(the potato late blight pathogen), *i.e*. in pathogen isolates showing the same allelic combination at ten microsatellite loci but polymorphic for virulence. Since French populations of *P. infestans *have been shown to be best adapted to the cultivar Bintje [24,25], which is free of known race-specific resistance genes, we measured the performance of isolates on this cultivar.

While Bahri et al. [23] performed pairwise competition experiments to assess isolates' fitness, we measured classical aggressiveness components (latent period, spore density and lesion growth rate) *in vitro *on detached leaflets, and aggregated them into a fitness measure corresponding to the *basic reproduction number *(R_0_) in mathematical and evolutionary epidemiology.

## Results

Among the 596 isolates tested, 162 different multilocus genotypes, corresponding to the presence/absence of each allele at each locus, were identified. The most frequent multilocus genotype (MLG; Table 1) was found in 132 isolates (22.1% of the collection). Because this clade showed a three-banded pattern at locus Pi63, its fingerprint cannot be analysed as if strictly diploid, preventing the use of software such as Genclone [26] to ascertain clonality. However, running Genclone on a subset of the collection (220 isolates) and using only the data from the eight polymorphic loci for which a maximum of two bands per isolate were detected showed that MLGs within this population were in all probability clones [27]. Therefore, the 132 isolates belonging to the most frequent MLG were considered as members of a quasi-isogenic clade, on which the study was performed.

**Table 1:** Allelic combination at each of ten microsatellite loci of a quasi-isogenic collection of *Phytophthora infestans *isolates chosen to measure fitness costs associated with unnecessary virulence factors.

Locus	PiG11	Pi56	Pi33	Pi63	Pi04	Pi70	Pi02	Pi4G	Pi16	Pi4B
**Alleles**	154/156	174/176	203	148/151/157	166/170	192/195	152/162	159	176/178	217

### Virulence

Virulence determination revealed 27 virulence phenotypes among the 132 quasi-isogenic isolates. The most common virulence profiles were 1-3-4-7-8-11 (*i.e*. isolates able to infect and sporulate on hosts possessing any combination of the race-specific resistance genes R1, R3, R4, R7, R8 and R11) (17%), 1-3-4-7-11 (17%), 1-3-4-7 (14%), 1-3-4-7-10-11 (10%), 1-3-4-7-8-10-11 (9%), 1-3-4-7-8 (7%), 1-3-4-7-10 (3%), 3-4-7 (3%) and 4-7 (2%). The other phenotypes were represented by only one or two isolates. Virulence phenotypes were usually complex, the mean number of virulence factors per isolate being 5. This allowed us to test the correlation between fitness and virulence complexity (ranging from two to eight virulence factors), and to compare the fitness of virulent and avirulent isolates for several virulence factors. That comparison was impossible for virulence to R2 and R5, because no isolate in the collection was virulent to either of these R-genes.

### Fitness costs

Fitness [as given by equation (1)] was computed for 1/μ = 80: the pathogen has 80 days to exploit a leaflet, which corresponds to the harvest or the natural leaflet death. The mean leaflet size X was set at 18 cm^2^, based on measures made on one hundred leaflets of the cultivar Bintje using a planimeter.

Isolates virulent to R10 were significantly less fit than avirulent isolates (Table 2). That fitness cost is due to a significantly lower spore density in virulent isolates; however, and unexpectedly, the latent period was significantly greater in avirulent isolates (Table 2). Whatever the R gene, lesion growth rates (and most often sporulation density) were lower and latency periods were shorter in virulent than in avirulent isolates. Although individual differences generally failed to reach statistical significance for genes other than R10 (Table 2), these distributions of differences for LGR and LP (χ^2 ^= 8.0, *P *= 0.005) as well as for SD (χ^2 ^= 2.0, *P *= 0.10), are unlikely to be due to chance only.

**Table 2:** Mean values of aggressiveness components (LP - latent period, days; SD - spore density, or number of spores per cm^2^; LGR - lesion growth rate, in cm^2 ^per day), as measured on the potato cultivar Bintje.

		**LP**	**SD**	**LGR**	**F**
		
**R1**	**Av (n = 20)**	3.38	25660	3.90	417888
	**V (n = 112)**	3.16	23369	3.32	378307
	**(F_1,130 _; P)**	9.44/0.0026**	1.59/0.2102	18.11/<.0001***	1.82/0.1792
		
**R3**	**Av (n = 13)**	3.28	23880	3.70	387640
	**V (n = 119)**	3.18	23698	3.38	383940
	**(F_1,130 _; P)**	1.37/0.2438	0.01/0.9342	3.45/0.0654	0.01/0.9172
		
**R4**	**Av (n = 5)**	3.37	24848	3.96	405237
	**V (n = 127)**	3.18	23671	3.39	383480
	**(F_1,130 _; P)**	1.81/0.1804	0.12/0.7326	4.58/0.0342*	0.15/0.6952
		
**R6**	**Av (n = 128)**	3.19	23742	3.42	384780
	**V (n = 4)**	3.04	22872	2.97	369069
	**(F_1,130 _; P)**	0.94/0.3331	0.05/0.8205	2.30/0.1315	0.06/0.7995
		
**R7**	**Av (n = 5)**	3.40	21453	3.80	348912
	**V (n = 127)**	3.18	23805	3.39	385698
	**(F_1,130 _; P)**	2.53/0.1141	0.47/0.4945	2.22/0.1388	0.44/0.5074
		
**R8**	**Av (n = 80)**	3.27	23661	3.58	384106
	**V (n = 52)**	3.08	23800	3.15	384609
	**(F_1,130 _; P)**	14.87/0.0002***	0.01/0.9179	18.19/<.0001***	0.00/0.9815
		
**R10**	**Av (n = 94)**	3.22	25054	3.47	406237
	**V (n = 38)**	3.11	20406	3.26	330051
	**(F_1,130 _; P)**	4.09/0.0453*	11.16/0.0011**	3.24/0.0741	11.57/0.0009***
		
**R11**	**Av (n = 50)**	3.25	25273	3.50	409722
	**V (n = 82)**	3.15	22766	3.36	368806
	**(F_1,130 _; P)**	3.39/0.0680	3.53/0.0626	1.82/0.1795	3.62/0.0594

Statistically significant differences are highlighted in bold and associated probabilities are indicated by stars (*: p < 0.05; **: p < 0.01; ***: p < 0.001).

Mean fitness values (F) for virulent and avirulent groups of quasi-isogenic *P. infestans *isolates with respect to different R-genes.

**Figure 1:**
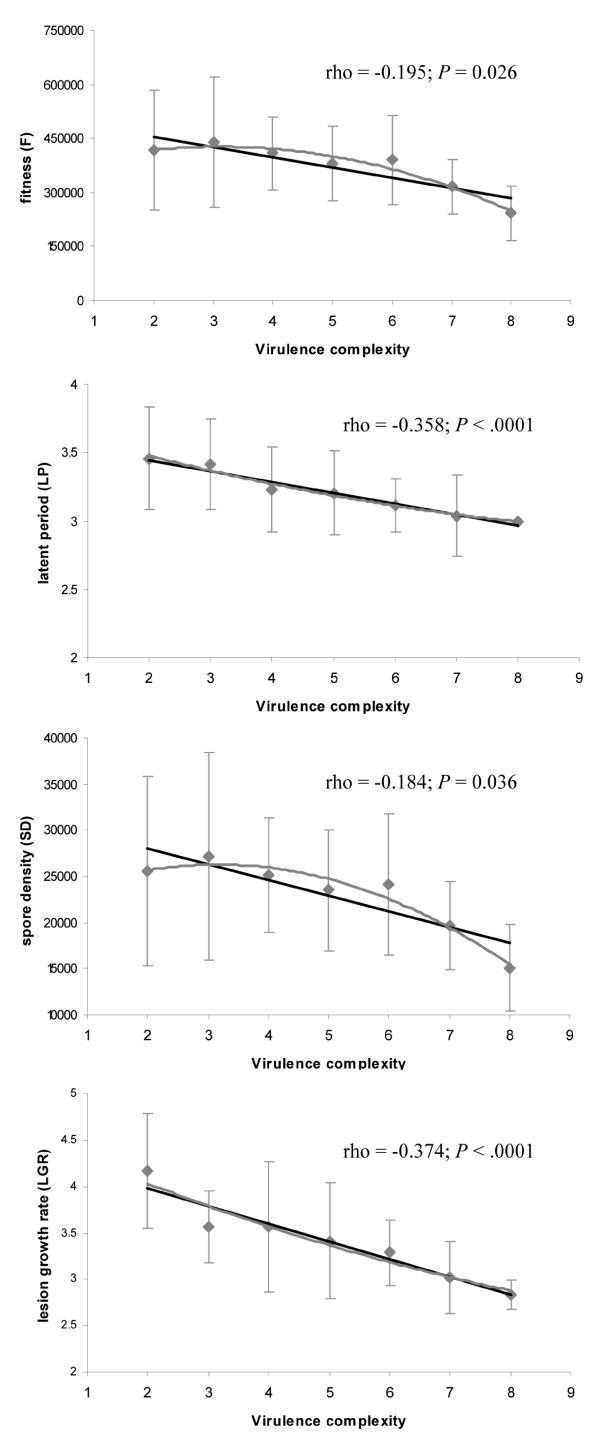
**Relationship between fitness, latent period, spore production and lesion growth rate (mean values ± SEM) and virulence complexity (*i.e*. the number of R-genes overcome by the isolate) for the 132 *P. infestans *isolates tested on the susceptible potato cultivar Bintje**. Spearman's rank correlation rho and corresponding P-values were indicated on the graphs. Linear and quadratic regression models were indicated in black and grey, respectively.

Fitness (F) and aggressiveness components (LP, SD and LGR) were negatively correlated with virulence complexity (Figure 1) indicating additive fitness impacts of virulence factors. Both linear and quadratic models fitted the data satisfactorily, whatever the considered variable (F, LP, SD and LGR). That is, the Akaike's information criterion (AIC) was lower for the linear model than for the quadratic one, yet this difference turned out not to be significant (data not showed).

## Discussion

The general objectives of this paper were to determine if fitness costs were associated with each of the main potato R-genes in *Phytophthora infestans*, and to identify the aggressiveness components concerned.

### Fitness costs: why so few?

A fitness cost was detected for R10, and specifically explained by a lower spore density in virulent isolates. However, virulent isolates showed a shorter latent period than avirulent isolates. This unexpected observation will be discussed below. Regarding the other virulence genes, the situation was less clear. A consistent trend nevertheless existed for virulent isolates sporulating less, growing slower but having a shorter latent period than avirulent ones. The fact that virulence complexity (the number of virulence factors an isolate possesses) correlates with fitness raised the issue of whether the test would have been significant if the virulent and avirulent sample sizes had been more evenly balanced.

On the other hand, it is also possible that there is no virulence cost associated with all R-genes except R10. This hypothesis is coherent with recent molecular data on the distribution of avirulence genes in the *P. infestans *genome: avirulence genes (with the signature RxLR motif) are present in very high copy numbers in the genome [28], so a mutated copy (virulent) could be associated with a non-mutated copy (avirulent) which could assure the initial function of the gene. This hypothesis is also consistent with population data. Indeed, if a gene imposes a fitness cost, its frequency should decrease in natural pathogen populations, at least when cultivars carrying the corresponding R gene are absent or withdrawn; conversely, virulent factors which are not costly can persist in populations. Andrivon [29] and Lebreton *et *al. [30] found the same complex virulence phenotypes (*i.e*. 1.3.4.7.8.11, 1.3.4.7.11 and 1.3.4.7) to dominate in *P. infestans *populations collected in French potato production areas, despite the absence of local selection by the corresponding R genes [31]. That stability of virulence phenotypes over a long period, at the French but also at a world-wide spatial scale [32,33], led to hypothesize that most phenotypes carry "fossil virulences" [34], which do not reflect local selection but selection in the pathogen's center of origin, prior to migration.

Another possibility, that Bahri et al. [23] also mentioned for the yellow rust pathogen *Puccinia striiformis*, is that virulence genes are not selected against because costs are compensated. Schoustra et al. [35], working with the filamentous ascomycete *Aspergillus nidulans*, found that fludioxonil-resistant isolates presented a severe fitness cost in the absence of the drug, but that this cost was afterwards waived without loss of the resistance. More generally, compensatory mutations have been detected, often in a fortuitous way, during genetic analyses of different viruses of animals or plants (see Luna et al. [36] and references therein).

### Fitness and aggressiveness components: an evolutionary explanation for an apparent biological contradiction?

There remains to be explained why virulent isolates often have a significantly lower latent period. A possible interpretation is the following. Let us assume the pathogen has to allocate host resources to either growth (mycelium) or reproduction (spores), and that possessing a virulence gene makes it more difficult to make mycelium. Then, reinvesting some resources otherwise allocated to mycelium into spore production would make sense, and would reduce the time to make a spore (the latent period). In other words, the shorter latent period in virulent isolates might well reflect an adaptive response from the pathogen to the cost of virulence. This question now merits to be analyzed in a more generic way, using both a theoretical modeling approach (*e.g*. [37]) and the analysis of experimental data from a range of pathosystems, including biotrophs, hemibiotrophs and necrotrophs.

### Fitness costs: small, but cumulative

A previous report on *P. infestans *indicated that race complexity (*i.e*. the number of R genes an isolate is able to overcome) had no effect on aggressiveness of isolates collected in Germany and in the Netherlands: laboratory and field tests did not show any decreased fitness of the more complex races compared to less complex races or to race 0 [38]. By contrast, our data, gathered on genetically related isolates, showed that fitness was negatively correlated with virulence complexity. This suggests that i) the small but consistent differences in aggressiveness observed for each individual gene are indeed cumulative, and may end-up in selection against the most complex races, and that ii) the rest of the pathogen genome (*i.e*. genes not directly related to pathogenicity) could be involved in the restoration of fitness costs due to unnecessary virulence.

Interestingly, a quadratic and concave regression model fitted the data as well as a linear model (Figure 1), suggesting that the marginal loss of fitness increases with every additional virulence. This finding, as observed earlier in a bacterial pathogen after serial knock-out of effector-coding genes [39], is consistent with theoretical multi-locus models explaining the persistence of polymorphisms in gene-for-gene interactions, and promotes diversity in resistance and avirulence genes [40]. It could thus explain the stability of polymorphisms in *P. infestans *populations, although these are dominated by clonal lineages.

Having such insights on the way virulent isolates respond to fitness costs may help making more durable the management of resistance genes. There is now both theoretical and experimental evidence supporting the idea to combine race-specific and quantitative resistance to enhance the durability of major genes [41-43]. Furthermore, we have shown that the fitness cost to the pathogen increases with the number of virulences, which could be an argument to build R-gene pyramids and benefit from a lower fitness of multi-virulent isolates (i.e. residual effects of defeated R-genes [44]). The cost of virulence to R10 could have been exploited in a such a pyramid of several R genes, or in genotypes combining R10 with high levels of partial resistance. Unfortunately, this is no longer possible, because R10 was originally introduced alone in susceptible genetic backgrounds, and has thus selected for virulent isolates that now constitute a very large fraction of the pathogen populations in most parts of the world (see for instance [32,33]). However, it points to the fact that race-specific resistance genes should be protected by such a combining strategy as soon as they are introduced into breeding material.

## Methods

### *Phytophthora infestans *biology

*P. infestans *belongs to the Oomycetes, a group of filamentous protists closely related to the brown algae [45]. *P. infestans *can infect all parts of the potato plant (*i.e*. shoots, stems, leaves, berries and tubers), leading to serious yield losses. It is characterized by a primarily aerial life cycle [46] with large multiplication rates [47] and polycyclic epidemics favoring rapid response to selection, which may however be counterbalanced by the large dispersion capacity of the asexual sporangia containing the infective zoospores [48]. *P. infestans *is a heterothallic species: sexual reproduction requires the simultaneous presence of hyphae of the two opposite mating types, designated A1 and A2 [49]. While infected tubers are the most common source of inoculum at the beginning of the season in temperate climates [50], infections can also start from oospores that result from the sexual cycle.

### Isolate collection

*Phytophthora infestans *isolates were collected during two consecutive years (2004 and 2005) in the two major French potato production areas, Brittany and northern France. Axenic cultures were established from 596 isolates. Each single-lesion isolate was obtained by placing 1 cm^2 ^pieces of infected tissue on tuber slices of potato cultivar Bintje, kept in plastic trays for six days at 15°C. Pure axenic cultures were then obtained by transferring small pieces of mycelium growing on the upper side of the potato slice to pea agar supplemented with 200 mg.L^-1 ^ampicillin, 30 mg.L^-1 ^rifamycin, and 0.4 ml.L^-1 ^pimaricin. After approximately 10 days at 15°C, growing colonies were transferred to pea agar without antibiotics, and subsequently maintained by serial transfers.

### Molecular characterization

Each of the 596 isolates of *P. infestans *was grown separately in pea broth autoclaved for 20 min at 120°C. After 10 to 15 days of incubation at 18°C, mycelium was washed three times in sterile water, and lyophilized. DNA was extracted as described by Lebreton et al. [30] and stored in TE buffer containing 10 M Tris-HCL and 0.1 M EDTA (pH 8.0). DNA concentration and purity were estimated using a spectrofluorimeter (SpectraMax M2).

Alleles at 10 polymorphic microsatellite loci - Pi4B, Pi4G and PiG11 developed by Knapova and Gisi [51]; and Pi02, Pi04, Pi16, Pi33, Pi56, Pi63 and Pi70 developed by Lees et al. [52] - were amplified in Polymerase Chain Reactions (PCR) performed in a 12.5 μL volume containing between 20 and 200 ng of DNA of *P. infestans*, 2.5 μL of 5× PCR Buffer (Promega), 0.3 mM of each dNTP, 2.5 mM of MgCl_2 _(Promega), 0.3 μM each of forward and reverse primers, and 1.25 U of Taq DNA polymerase (GoTaq^® ^flexi DNA polymerase, Promega). PCRs were performed in a MJ Research thermocycler under the following conditions: each reaction started with a cycle of 2 min at 95°C, followed by 30 cycles of 20 s at 95°C, 25 s at 56°C (for PiG11), 58°C (for Pi02, Pi04, Pi16, Pi33, Pi56, Pi63, Pi70 and Pi4B) or 60°C (for Pi4G) and 60 s at 72°C, and finished with an elongation cycle of 5 min at 72°C. In order to detect simultaneously the alleles at several loci, primers were labeled with one of three fluorescent dyes: FAM (PiG11, Pi33, Pi 63, Pi70, Pi02, and Pi4B), NED (Pi56, Pi04, Pi4G) and HEX (Pi16). Amplification products were pooled into three groups, based on expected allele sizes: PiG11, Pi56 and Pi33; Pi63, Pi04, and Pi70; and Pi02, Pi4G, Pi16 and Pi4B, respectively. Ten μL samples, comprising 9.84 μL of deionized formamide Hi-Di™ (Applied Biosystems), 0.06 μL of 400 HD ROX™ Size standard (Applied Biosystems), and 0.1 μL of PCR multiplexed product, were loaded into an ABI Prism 3130*xl *DNA sequencer run according to manufacturer's instructions (Applied Biosystems). DNA fragments were automatically sized with the GeneMapper™ 3.5 software. Allele sizes were calibrated to the allele sizes of reference isolates kindly provided by Drs Lees and Cooke of the Scottish Crop Research Institute, UK [52].

### Pathogenicity tests

#### Potato plant material

Plants of the potato cultivar Bintje were grown from certified seed tubers in 13 cm pots (one tuber per pot) filled with 1:1:1 sand-peat-compost mixture, in a glasshouse regulated at 15-20°C (night/day temperatures) and 16 h of photoperiod. Plants were watered with a nutrient solution (Hakaphos; NPK 15/10/15) once a week.

#### Inoculum preparation

Sporangial suspensions of each of the 132 isolates of the quasi-isogenic collection were prepared from 20-days-old cultures on pea agar, and adjusted to 5.10^4 ^sporangia per mL using a haemacytometer. Before inoculation, sporangial suspensions were kept at 4°C for approximately 4 hours to promote zoospore liberation.

#### Virulence phenotype determination

Virulence patterns were determined using the international late blight differential set of potato clones, each having one of the R1-R11 race-specific resistance genes, and 2 susceptible cultivars (Bintje and Craigs Royal). Recent work has shown that the R3 differential (CEBECO-4642-1) actually contains two closely linked genes, R3a and R3b [53]. Virulence to each of these could not be assessed separately, due to the lack of available differential hosts with only one of these two genes. Therefore, virulence to R3 was regarded as a single factor. Virulence to R9 could not be assessed because the corresponding differential clone was not present in our test set; to our knowledge, this gene has never been introduced into commercial European cultivars. Since the physiological age of plants can affect the expression of resistance genes [54], all tests were performed using leaflets detached from 6-8-week-old plants. Each leaflet was placed abaxial face up on a moist filter paper in a clear plastic dish, and inoculated by depositing a 20 μL drop of the sporangial suspension (containing *ca*. 1000 spores) on each side of the midrib. Two leaflets per isolate and differential host were inoculated. Dishes containing the inoculated leaflets were deposited in clear plastic boxes, and incubated in an illuminated incubator for 7 days at 18°C/15°C day/night with 16 h day length. After incubation, each inoculation site was scored for the presence or absence of a sporulating lesion. This method has proved reliable to assess virulence of a range of diversified isolates in a ring test across 12 European laboratories [55].

#### Aggressiveness quantification

Each isolate was tested for aggressiveness on the cultivar Bintje. Six leaflets were detached from 6-8-week-old plants, and placed abaxial face up on the lids of inverted Petri dishes containing 12 g.L^-1 ^water agar (two leaflets per dish). Each leaflet was inoculated by depositing a 20 μL drop of the sporangial suspension as close as possible to the leaflet centre. Inoculated leaflets were then incubated as described for the virulence tests. The *latent period *(LP) was determined by observing daily the appearance of sporangia. Seven days after inoculation, lesion diameters were measured in two perpendicular directions and lesion size was computed assuming an elliptic shape. Then, leaflets were washed in 10 mL Isoton II (saline buffer), and the sporangia production per leaflet was determined with a Coulter Z2 counter (Beckman Coulter France, Villepinte, France). This allowed measuring the *spore density *(SD), *i.e*. the number of sporangia produced per cm^2 ^of lesion, and assessing the *lesion growth rate *(LGR), assumed constant in cm^2 ^per day [56]. More accurately, the lesion size was divided by the time elapsed since it started growing, at time LP (the lesion represents the area having sporulated).

### Fitness estimation

An epidemiological model was developed to derive the *basic reproduction numbe*r of the pathogen (R_0_, equal to the number of secondary infections generated by each lesion). This allowed to aggregate measures made on leaflets (LP, SD and LGR) into a single fitness estimate.

The model is the following. Let S, E and I denote the densities of susceptible (healthy), exposed (or latent, *i.e*. infected but not yet infectious) and infectious (*i.e*. sporulating) hosts (or leaflets), respectively. Let also the prime denote differentiation with respect to time (e.g. S' = dS/dt). We then consider the classical SEIR model (see for instance [57] for an introduction to SIR-like modeling):

S'=B−βSI−μS,E'=βSI−E/λ−μE,I'=E/λ−αI−μI,

where B is the "birth" rate and 1/*μ *represents the time available to the pathogen to exploit a leaflet (since harvest or natural death make it finite). The three epidemiological parameters, denoted *β*, *λ *and *α *are defined as the transmission rate, the latent period and the inverse of the infectious period, respectively. Clearly, *λ *= LP. Let X be the underlying leaflet size (a constant) in this model. Since *α *is the inverse of the time actually taken to exploit a leaflet after the latent period has elapsed, *α *= LGR/X. To estimate *β*, let Y be the probability for a released spore to come in contact with a particular host times the infection success rate. Then, *β *= Y. *σ*, where *σ *is the number of sporangia produced per unit time per infectious host (see Madden et al. [58]), that is, *σ *= SD.LGR.

According to van den Driessche & Watmough [59], the basic reproductive number in this SEI model is:

$${\text{R}}_{\text{0}}=\frac{1}{1+\lambda .\mu }\frac{\beta \text{​}S_{0}}{\alpha \;+\;\mu },$$

where S_0 _is the healthy host density at the disease-free equilibrium. Proceeding as in Diekmann [60], an appropriate fitness measure can thus be defined as

(1) $$F=\frac{1}{1+\lambda .\mu }\frac{\sigma }{\alpha \;+\;\mu }=\frac{1}{1+LP.\mu }\frac{SD.LGR}{\frac{LGR}{X}+\mu }\;.$$

### Data analyses

The effect of virulence/avirulence (vir/avir effect) on the aggressiveness components (LP, SD and LGR) and on the fitness (F) distributions was tested for each R-gene through a one-way ANOVA using the GLM procedure of the SAS statistical software, v. 8.1 (SAS Institute, Cary, NC). The Shapiro-Wilk test showed that residuals have a normal distribution. The fitness value was calculated for each of the 132 isolates from the experimental data, using fixed values of X (18 cm^2^, mean leaflet size in cv Bintje) and 1/μ (80 days, time available to the pathogen to exploit a leaflet since harvest or natural death). Additionally, the relationship between fitness (F) or aggressiveness components (LP, SD and LGR) and *virulence complexity *(*i.e*. the number of R-genes overcome by the isolate) was tested with the Spearman's rank correlation rho using the statistical freeware R, version 2.9.2 (R Development Core Team 2009), to detect compensatory or additive effects of costs from different virulence factors. The model selection based on Akaike's information criteria, AIC [61], was used in order to determine which parameterized model (linear or quadratic) best fitted the data.

## Authors' contributions

JM, IG, and RC performed the experiments according to a protocol elaborated jointly between JM and DA. FMH introduced the "basic reproductive number" from Evolutionary Epidemiology as a fitness measure. JM and FMH analyzed the data. JM, FMH and DA wrote the text and prepared the figures. All authors read and approved the final manuscript.
